# Can toothbrushing reduce the intraoral viral load of SARS-CoV-2? A pilot study with a dentifrice containing an antimicrobial phthalocyanine derivative

**DOI:** 10.3205/dgkh000487

**Published:** 2024-06-05

**Authors:** Marcelo Lupion Poleti, Danielle Gregório, Alisson Gabriel Idelfonso Bistaffa, Fabiano Vieira Vilhena, Andréa Name Colado Simão, Mayara Tiemi Enokida Mori, Nicole Perugini Stadtlober, Marcell Alysson Batisti Lozovoy, Paulo Sérgio da Silva Santos, Berenice Tomoko Tatibana, Thais Maria Freire Fernandes

**Affiliations:** 1 Oral Health, Federal Institute of Paraná – IFPR, Londrina, Brazil; 2 Postgraduate Program in Dentistry, School of Dentistry, University of North Paraná – UNOPAR/UNIDERP, Londrina, Brazil; 3 TRIALS – Oral Health & Technologies, Bauru, Brazil; 4 Research Laboratory in Applied Immunology, Department of Pathology, Clinical Analysis and Toxicology, State University of Londrina, Londrina, Brazil; 5 Department of Surgery, Stomatology, Pathology, and Radiology, Bauru School of Dentistry, University of São Paulo, Bauru, Brazil

## Abstract

The aim of this study was to assess whether toothbrushing with a dentifrice containing an antimicrobial phthalocyanine derivative (APD) can reduce the intraoral viral load of SARS-CoV-2. Twenty COVID-19-positive dentate patients aged ≥18 years were selected instructed to brush their teeth for 2 min with a dentifrice containing APD. Self-collected samples of unstimulated saliva were carried out three times: T0 (baseline), T5 (5 min after toothbrushing), and T30 (30 min after toothbrushing). The analysis of viral RNA was performed by RT-qPCR for detection of three viral genes (ORF1ab, N and S genes). Results were statistically tested using Friedman’s test and pairwise comparison with Bonferroni corrections, with a significance level of 5%. There was an increase in the cycle threshold (Ct) value from T0 to T5 in 13 patients (72.2%), and from T0 to T30 in 14 patients (77.8%). In two patients (11.1%) no SARS-CoV-2 was detected at T5 and five patients (27.8%) at T30. The Ct values were statistically significantly higher (p=0.020) at T30 in comparison to T0 and T5.

This pilot study suggests that toothbrushing with a dentifrice containing APD could reduce the SARS-CoV-2 viral load in the oral cavity. However, further studies are needed to confirm this possible beneficial effect against SARS-CoV-2.

## Abstract (de)

Es sollte untersucht werden, ob Zähneputzen mit einer Zahnpasta, die ein antimikrobielles Phthalocyanin-Derivat (APD) enthält, die intraorale Viruslast von SARS-CoV-2 verringern kann. 20 COVID-19-positive Zahnpatienten im Alter von 18 Jahren oder älter wurden ausgewählt und mussten sich 2 min lang mit einer APD-haltigen Zahnpasta die Zähne bürsten.

Proben von unstimuliertem Speichel wurden von den Probanden dreimal selbst entnommen: T0 (Ausgangswert), T5 (5 min nach dem Zähneputzen) und T30 (30 min nach dem Zähneputzen). Die Analyse der viralen RNA wurde mittels RT-qPCR zum Nachweis von drei viralen Genen (ORF1ab, N und S) durchgeführt. Verwendet wurde der Friedman-Test und der paarweise Vergleich mit Bonferroni-Korrektur mit einem Signifikanzniveau von 5% v Bei 13 Patienten (72,2%) stieg der Zyklus-Schwellenwert Ct von T0 auf T5 und bei 14 Patienten (77,8%) von T0 auf T30 an. Bei zwei Patienten (11,1%) wurde bei T5 kein SARS-CoV-2 nachgewiesen und bei fünf Patienten (27,8%) bei T30. Die Ct-Werte waren bei T30 im Vergleich zu T0 und T5 signifikant höher (p=0,020).

Die Pilotstudie deutet darauf hin, dass Zähneputzen mit einer APD-haltigen Zahnpasta die SARS-CoV-2-Viruslast in der Mundhöhle verringern könnte. Es sind jedoch weitere Studien erforderlich, um diese mögliche positive Wirkung gegen SARS-CoV-2 zu bestätigen.

## Introduction

On March 11, 2020, the World Health Organization (WHO) declared Coronavirus Disease 2019 (COVID-19) as a pandemic, a worldwide catastrophe, and nearly 5 million deaths have been reported since then [1], [2], [3]. Airborne contamination, respiratory droplets and direct contact are the main sources of infection with SARS-CoV-2 [2], [4], [5]. Measures to prevent the spread of COVID-19 have been used, e.g., mask-wearing, social distancing and hand antisepsis [6], with an emphasis on vaccines. Since the mouth is involved in the pathophysiology of COVID-19 [7], [8], [9], [10] and given the relationship between oral health and disease severity [8], [9], dental care has become even more important [11], [12], [13], [14]. In this pandemic context, the use of adjuvant preventive measures, such as toothbrushing, gargling, rinsing and the use of oral hygiene products, has been reported [11], [12], [15], [16], [17], [18], [19], [20]. Recently, an antimicrobial phthalocyanine derivative (APD) compound was incorporated in oral hygiene products [16], [19], [20], [21], [22], [23], [24]. Two previous studies have shown the beneficial effects of an APD mouthwash gargling/rinsing protocol in COVID-19 patients, such as rapid amelioration of sore throats, cough, mouth ulcers and a significant decrease in the length of hospitalization [19], [20]. Additionally, an *in-vitro* study demonstrated 90% and 99–99% SARS-CoV-2 inactivation with an oral rinse and a dentifrice containing APD, respectively [21]. However, there are no results from clinical studies on the effect of toothbrushing with dentifrice containing APD on the intraoral viral load of SARS-CoV-2.

In the current pilot study, we assessed whether toothbrushing with a dentifrice containing APD can reduce the intraoral viral load of SARS-CoV-2-positive subjects.

## Materials and methods

### Ethics

This project was carried out in compliance with relevant laws and guidelines, and with the ethical standards of the Declaration of Helsinki. It was approved by the Ethics Committee of the Federal Institute of Paraná (CAAE 35194520.0.0000.8156) upon permission of the Londrina Municipal Health Authority. 

### Study design and subjects

The present work was designed as a cross-sectional clinical pilot study to assess whether toothbrushing with a dentifrice containing the antimicrobial agent APD can reduce the intraoral viral load of SARS-CoV-2.

Taken as a convenience sample, the subjects consisted of 20 dentate adult patients of both genders aged ≥18 years who lacked comorbidities and were non-smokers, who had mild symptoms and were diagnosed with COVID-19 by real-time reverse transcription-polymerase chain reaction (RT-PCR) in nasopharyngeal swab samples at a reference center for the diagnosis of COVID-19 in Londrina, Brazil.

An online questionnaire was sent to collect the demographic characteristics of the patients and clinical data about COVID-19 symptoms using the Mentimeter system (Mentimeter AB, Stockholm, Sweden).

### Samples collection

After telephone contact and agreement to participate in the research, the researchers took a kit containing three 15-ml falcon tubes (Corning Incorporated, USA), a dentifrice containing APD (DentalClean, Rabbit Corp, Londrina, Brazil), and a toothbrush (DentalClean, Rabbit Corp, Londrina, Brazil) to the residence of each volunteer. Videos with instructions for performing the saliva self-collection and toothbrushing were sent via WhatsApp.

The self-collected samples of unstimulated saliva were performed in the morning, before breakfast. Saliva collection was carried out three times in the same day: T0 (baseline, before toothbrushing); T5 (5 min after toothbrushing); and T30 (30 min after toothbrushing). The patients did not eat or drink and or use any oral hygiene product or medication during the entire collection period (30 min). For acquiring a baseline saliva specimen (T0) for the SARS-CoV-2, participants were asked to rinse their mouths with 5 ml of water, then all the saliva produced was poured into the tube for 10 min. Immediately afterwards, they were instructed to use the same amount of dentifrice to brush their teeth and tongue for 2 min. After 5 and 30 min of toothbrushing, the saliva collection procedure was repeated.

The samples were stored in freezers (–20ºC) until a special service arrived to transport them to the COVID-19 testing laboratory. There, the samples were stored at –80ºC until analysis.

### RNA extraction of SARS-CoV-2 using magnetic beads

Viral RNA was extracted from 100 µL of saliva collected at each time period, using the automatized extractor EXTRACTA 32 (LOCCUS, Cotia, Brazil) and magnetic-bead extraction kits (MVXA-P016 FAST), following the manufacturer’s instructions (LOCCUS, Cotia, Brazil). A negative extraction control (UltraPure™ DNase/RNase-Free Distilled Water, Thermo Fisher Scientific, Waltham, MA, USA) was added to each extraction run. 

### SARS-CoV-2 RNA detection by RT-qPCR 

The qualitative analysis of viral RNA was performed by RT-PCR using the TaqPath™ COVID-19 multiplex Real-Time RT-PCR (RT-qPCR) test for detection of three viral genes (ORF1ab, N and S genes) (Thermo Fisher Scientific, Waltham, MA, USA) according to the manufacturer’s instructions. Positive and negative controls were analyzed simultaneously with the samples. All stages of RT-PCR, including cDNA synthesis and amplification of the target sequences, were performed in the QuantStudio™ 6 FLEX Real-Time PCR system (Thermo Fisher Scientific, Waltham, MA, USA). Results were considered positive when the cycle threshold (Ct) values were ≤37 for two or more genes and negative when the Ct values were >37 for three SARS-CoV-2 targets (ORF1ab, N, and S genes). 

This was the same methodology used to diagnose COVID-19 from nasopharyngeal swab samples and was performed in the same laboratory by the same team of technicians.

### Statistical analysis

The evaluation of the effects was based on differences in Ct values. The medians of the Ct values of the three genes (ORF1ab, N, and S) were calculated to avoid the influence of the outliers in the data set and were used for statistical analysis. The Kolmogorov-Smirnov test was used to assess the normality of the variables. Continuous variables were described with median values with interquartile range (IQR) for non-normally distributed variables, and with mean ± standard deviation (SD) values for mean age (a normally distributed variable). Friedman's test and pairwise comparison with Bonferroni corrections were used to compare the differences in the Ct values between the groups. The infectivity was classified according to the Ct value obtained: high viral load (Ct<25), intermediate viral load (Ct: 25–30) and low viral load (Ct>30) [25]. Statistical significance was set at p<0.05. All statistical analyses were performed using IBM SPSS Statistics, version 27 (IBM Corp., Armonk, NY, USA).

## Results

Twenty patients were initially included in this study, but two patients were excluded because no SARS-CoV-2 could be detected in the saliva specimen. The patient characteristics are shown in Table 1 (Tab. 1). The 18 patients (8 female, 10 male) had a mean age of 30.6 years (SD: 8.50). No adverse events were reported by any of the patients. The median period between onset of symptoms and swab collection was 4 days (IQR: 3–6). According to the viral load on swabs, the median Ct value was 19.8 (IQR: 18.9–21.1). Seventeen patients (94.4%) had a high viral load and one patient (5.6%) an intermediate viral load. The median period between onset of symptoms and toothbrushing/saliva collections was 8 days (IQR: 7–10), and the baseline saliva Ct value was 29.6 (IQR: 22.1–32.3). 

### Analysis of Ct value before and after toothbrushing

**Figure 1:**
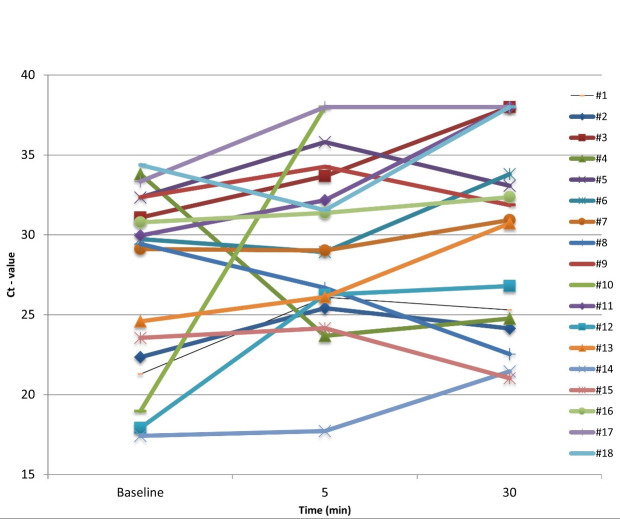
Ct values of detection of SARS-CoV-2 genes in saliva at T0 (baseline), T5 and T30

Figure 1 (Fig. 1) shows the Ct values of detection of SARS-CoV-2 genes in saliva at T0, T5 and T30. There was an increase in the Ct value from T0 to T5 in 13 patients (72.2%), and from T0 to T30 in 14 patients (77.8%). In two patients (11.1%). no SARS-CoV-2 was detected at T5, increasing to five patients (27.8%) at T30.

The Ct values were significantly higher (p=0.020) at T30 in comparison to T0 and T5 (Table 2 (Tab. 2)). The greatest difference in the Ct values was between T30 and T0 (3.8).

## Discussion

The pilot study demonstrated intraoral reduction of the SARS-CoV-2 viral load after toothbrushing using a dentifrice containing APD. In each of the 18 subjects (who served as their own controls), the intraoral viral load was examined in the saliva at baseline (T0) and 5 and 30 min after toothbrushing, and the patients themselves carried out the saliva collections at home. This methodology is in accordance with Valentine-Graves et al. [26], who concluded that at-home self-collection makes it possible to reduce the individual’s exposure, the need for personal protective equipment/cost, and also offers options for screening populations without symptoms.

Understanding the pathophysiology of COVID-19, with SARS-CoV2 having its affinity for the mucous membranes of the mouth and oropharynx, and the salivary glands as a reservoir, lends research such as this study strategic importance in the global fight against COVID-19 [27], [28]. A recent study by Huang et al. [28] confirmed SARS-CoV-2 infection in the salivary glands and oral mucosa by identifying the host entry factors (ACE2 and TMPRSS). The authors reported that salivary glands and oral mucosa could play an important role in transmitting the SARS-CoV-2 to the lungs or the gastrointestinal tract via saliva. Huang et al. concluded that the oral cavity is an important site for SARS-CoV-2 and saliva as a potential route of COVID-19 transmission from oral droplets containing infectious virus and infected cells. A study by Matuck et al. [10] demonstrated the presence of SARS-CoV-2 in periodontal tissue in severely ill patients. Those authors highlight that periodontal tissue can be a target for SARS-CoV-2 and contribute to the presence of the virus in saliva. Therefore, under these circumstances, all aspects of oral hygiene are crucial areas for preventing the spread of the virus; the use of antiviral oral-care products could be an adjuvant against the SARS-CoV-2 [29]. In this sense, the WHO [30] and the German Society of Hospital Hygiene [31], [32] recommend that dental practices routinely ask patients to decontaminate their oral cavity with an antiviral mouthwash prior to examination.

The results of the present study found that after 30 min of toothbrushing, the Ct values in the saliva of 77.8% of the patients increased. Of these, no SARS-CoV-2 was detected at T30 in 27.8% (Figure 1 (Fig. 1)), which may have substantially reduced the infectivity of disease. The estimate of viral load reduction was based on the mean increase in the Ct values of 3.8 units, which may correspond to at least a 10-fold less target RNA [33]. Previous studies have shown that Ct values offer a semi-quantitative analysis of viral RNA concentration, i.e., lower Ct values correspond to higher viral RNA concentrations, and can serve as an indirect indicator of the relative viral load of SARS-CoV-2 [33], [34].

The limitations of this study were that no virus cultures were performed for SARS-CoV-2, substantivity analysis of the dentifrice after 30 min was not performed, there was no toothbrushing group without APD, and no control group without toothbrushing.

We believe that the sum of the mechanical action associated with the antiviral action of toothbrushing could be a useful adjuvant in the fight against the pandemic. The results of this pilot study suggest the need for further, prospective studies evaluating the effectiveness of products with antiviral characteristics and the mechanical actions of reducing oral microbiota. Such studies should involve longer periods of use, as well as the analysis of substantivity for a longer period before determining the antiviral effect.

## Conclusion

The pilot study suggests that toothbrushing with a dentifrice containing APD could reduce the SARS-CoV-2 viral load in the oral cavity. However, further studies are needed to confirm this possible beneficial effect against SARS-CoV-2.

## Notes

### Acknowledgement 

The authors gratefully acknowledge the Londrina Municipal Health Board for supporting this study. 

### Competing interests

Dr. Vilhena has a patent pending. The other authors declare that they have no competing interests.

### ORCID of the author

Marcelo Lupion Poleti: 0000-0003-1904-5762

## Figures and Tables

**Table 1:**
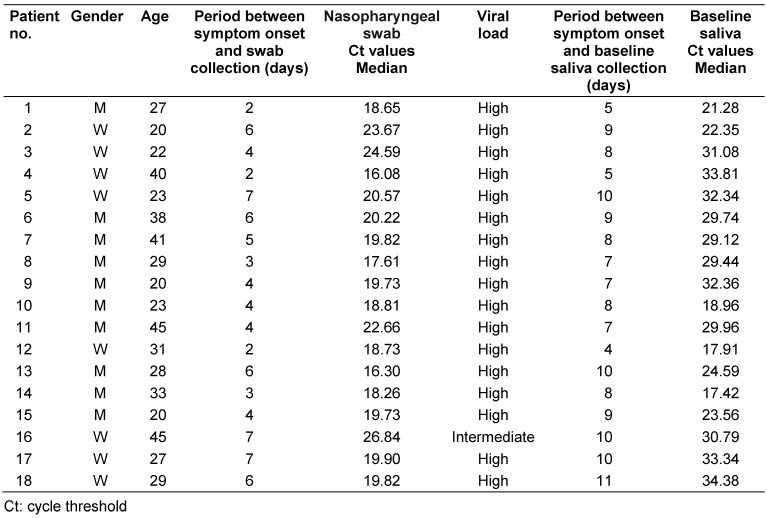
Characteristics of patients with SARS-CoV-2 detected in the nasopharyngeal swab and baseline saliva by RT-qPCR

**Table 2:**
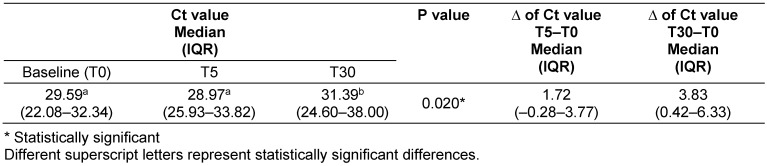
Comparison of the Ct value in saliva between baseline, T5 and T30 groups

## References

[1] Cucinotta D, Vanelli M (2020). WHO Declares COVID-19 a Pandemic. Acta Biomed.

[2] Habas K, Nganwuchu C, Shahzad F, Gopalan R, Haque M, Rahman S, Majumder AA, Nasim T (2020). Resolution of coronavirus disease 2019 (COVID-19). Expert Rev Anti Infect Ther.

[3] World Health Organization (WHO) (2022). COVID-19 Weekly Epidemiological Update.

[4] van Doremalen N, Bushmaker T, Morris DH, Holbrook MG, Gamble A, Williamson BN, Tamin A, Harcourt JL, Thornburg NJ, Gerber SI, Lloyd-Smith JO, de Wit E, Munster VJ (2020). Aerosol and Surface Stability of SARS-CoV-2 as Compared with SARS-CoV-1. N Engl J Med.

[5] Mersha A, Shibiru S, Girma M, Ayele G, Bante A, Kassa M, Abebe S, Shewangizaw M (2021). Perceived barriers to the practice of preventive measures for COVID-19 pandemic among health professionals in public health facilities of the Gamo zone, southern Ethiopia: a phenomenological study. BMC Public Health.

[6] World Health Organization (WHO) (2020). Mask use in the context of COVID-19: interim guidance, 1 December 2020.

[7] Huang N, Pérez P, Kato T, Mikami Y, Okuda K, Gilmore RC, Conde CD, Gasmi B, Stein S, Beach M, Pelayo E, Maldonado JO, Lafont BA, Jang SI, Nasir N, Padilla RJ, Murrah VA, Maile R, Lovell W, Wallet SM, Bowman NM, Meinig SL, Wolfgang MC, Choudhury SN, Novotny M, Aevermann BD, Scheuermann RH, Cannon G, Anderson CW, Lee RE, Marchesan JT, Bush M, Freire M, Kimple AJ, Herr DL, Rabin J, Grazioli A, Das S, French BN, Pranzatelli T, Chiorini JA, Kleiner DE, Pittaluga S, Hewitt SM, Burbelo PD, Chertow D, NIH COVID-19 Autopsy Consortium, HCA Oral and Craniofacial Biological NetworkFrank K, Lee J, Boucher RC, Teichmann SA, Warner BM, Byrd KM (2021). SARS-CoV-2 infection of the oral cavity and saliva. Nat Med.

[8] Iranmanesh B, Khalili M, Amiri R, Zartab H, Aflatoonian M (2021). Oral manifestations of COVID-19 disease: A review article. Dermatol Ther.

[9] Kamel AHM, Basuoni A, Salem ZA, AbuBakr N (2021). The impact of oral health status on COVID-19 severity, recovery period and C-reactive protein values. Br Dent J.

[10] Fernandes Matuck B, Dolhnikoff M, Maia GVA, Isaac Sendyk D, Zarpellon A, Costa Gomes S, Duarte-Neto AN, Rebello Pinho JR, Gomes-Gouvêa MS, Sousa SCOM, Mauad T, Saldiva PHDN, Braz-Silva PH, da Silva LFF (2020). Periodontal tissues are targets for Sars-Cov-2: a post-mortem study. J Oral Microbiol.

[11] Alharbi A, Alharbi S, Alqaidi S (2020). Guidelines for dental care provision during the COVID-19 pandemic. Saudi Dent J.

[12] Sampson V, Kamona N, Sampson A (2020). Could there be a link between oral hygiene and the severity of SARS-CoV-2 infections? Br Dent J.

[13] Kumar U, Gupta A, Goyal A, Gauba K (2021). Impact of covid-19 pandemic on characteristics of dental emergencies and treatment services at tertiary care centre. Saudi Dent J.

[14] Pinzan-Vercelino CR, Freitas KM, Girão VM, da Silva DO, Peloso RM, Pinzan A (2021). Does the use of face masks during the COVID-19 pandemic impact on oral hygiene habits, oral conditions, reasons to seek dental care and esthetic concerns? J Clin Exp Dent.

[15] González-Olmo MJ, Delgado-Ramos B, Ruiz-Guillén A, Romero-Maroto M, Carrillo-Díaz M (2020). Oral hygiene habits and possible transmission of COVID-19 among cohabitants. BMC Oral Health.

[16] da Orcina B, Vilhena FV, Cardoso de Oliveira R, Marques da Costa Alves L, Araki K, Hiroshi Toma S, Schutzer Ragghianti Zangrando M, da Silva Santos PS (2020). PHTALOX® Mouthwash as An Option to Reduce Clinical Symptoms of COVID-19: Case Series (November 13, 2020) [Preprint]. SSRN.

[17] Carrouel F, Valette M, Gadea E, Esparcieux A, Illes G, Langlois ME, Perrier H, Dussart C, Tramini P, Ribaud M, Bouscambert-Duchamp M, Bourgeois D (2021). Use of an antiviral mouthwash as a barrier measure in the SARS-CoV-2 transmission in adults with asymptomatic to mild COVID-19: a multicentre, randomized, double-blind controlled trial. Clin Microbiol Infect.

[18] Chopra A, Sivaraman K, Radhakrishnan R, Balakrishnan D, Narayana A (2021). Can povidone iodine gargle/mouthrinse inactivate SARS-CoV-2 and decrease the risk of nosocomial and community transmission during the COVID-19 pandemic? An evidence-based update. Jpn Dent Sci Rev.

[19] da Silva Santos PS, da Fonseca Orcina B, Machado RRG, Vilhena FV, da Costa Alves LM, Zangrando MSR, de Oliveira RC, Soares MQS, Simão ANC, Pietro ECIN, Kuroda JPG, de Almeida Benjamim IA, Araujo DB, Toma SH, Flor L, Araki K, Durigon EL (2021). Beneficial effects of a mouthwash containing an antiviral phthalocyanine derivative on the length of hospital stay for COVID-19: randomised trial. Sci Rep.

[20] da Fonseca Orcina B, Vilhena FV, Cardoso de Oliveira R, Marques da Costa Alves L, Araki K, Toma SH, Ragghianti Zangrando MS, da Silva Santos PS (2021). A Phthalocyanine Derivate Mouthwash to Gargling/Rinsing as an Option to Reduce Clinical Symptoms of COVID-19: Case Series. Clin Cosmet Investig Dent.

[21] Santos C, da Fonseca Orcina B, Brito Reia VC, Ribeiro LG, Grotto RMT, Prudenciatti A, de Moraes LN, Ragghianti Zangrando M, Vilhena FV, da Silva Santos PS (2021). Virucidal Activity of the Antiseptic Mouthwash and Dental Gel Containing Anionic Phthalocyanine Derivative: In vitro Study. Clin Cosmet Investig Dent.

[22] Teodoro G, Santos C, Carvalho M, Koga-ito C, Sibelino S, Vilhena F PHTALOX® antimicrobial action and cytotoxicity: in vitro study [Abstract]. https://iadr.abstractarchives.com/abstract/20iags-3314903/phtalox-antimicrobial-action-and-cytotoxicity-in-vitro-study.

[23] Brito-Reia VC, da Silva Bastos R, Vieira Vilhena F, Marques Honório H, Marques da Costa Alves L, Frazão P, Sérgio da Silva Santos P (2022). Population-based virucidal phthalocyanine gargling/rinsing protocol to reduce the risk of coronavirus disease-2019: a community trial. GMS Hyg Infect Control.

[24] da Fonseca Orcina B, Reia VCB, Simão ANC, Lonni AASG, Fernandes TMF, Poleti ML, Vilhena FV, da Silva Santos PS (2022). A recommendation of PHTALOX® for preventing infection and progression of COVID-19: a 1-year summarized update of scientific approaches. GMS Hyg Infect Control.

[25] de la Calle C, Lalueza A, Mancheño-Losa M, Maestro-de la Calle G, Lora-Tamayo J, Arrieta E, García-Reyne A, Losada I, de Miguel B, Díaz-Simón R, López-Medrano F, Fernández-Ruiz M, Carretero O, San Juan R, Aguado JM, Lumbreras C (2021). Impact of viral load at admission on the development of respiratory failure in hospitalized patients with SARS-CoV-2 infection. Eur J Clin Microbiol Infect Dis.

[26] Valentine-Graves M, Hall E, Guest JL, Adam E, Valencia R, Shinn K, Hardee I, Sanchez T, Siegler AJ, Sullivan PS (2020). At-home self-collection of saliva, oropharyngeal swabs and dried blood spots for SARS-CoV-2 diagnosis and serology: Post-collection acceptability of specimen collection process and patient confidence in specimens. PLoS One.

[27] Jafer MA, Hazazi MA, Mashi MH, Sumayli HA, Mobarki YJA, Sultan A, Ali Hadi MS, Abulqasim HM, Thubab BMM, Patil S (2020). COVID-19 and Periodontitis: A Reality to Live with. J Contemp Dent Pract.

[28] Huang YH, Huang JT (2021). Use of chlorhexidine to eradicate oropharyngeal SARS-CoV-2 in COVID-19 patients. J Med Virol.

[29] de Toledo Telles-Araujo G, Caminha RDG, Kallás MS, Sipahi AM, da Silva Santos PS (2020). Potential mouth rinses and nasal sprays that reduce SARS-CoV-2 viral load: What we know so far? Clinics (Sao Paulo).

[30] World Health Organization (WHO) (2020). Considerations for the provision of essential oral health services in the context of COVID-19: interim guidance, 3 August 2020.

[31] Kramer A, Eggers M, Hübner NO, Walger P, Steinmann E, Exner M (2021). Virucidal gargling and virucidal nasal spray. GMS Hyg Infect Control.

[32] Kramer A, Eggers M, Exner M, Hübner NO, Simon A, Steinmann E, Walger P, Zwicker P (2022). Recommendation of the German Society of Hospital Hygiene (DGKH): Prevention of COVID-19 by virucidal gargling and virucidal nasal spray - updated version April 2022. GMS Hyg Infect Control.

[33] Tom MR, Mina MJ (2020). To Interpret the SARS-CoV-2 Test, Consider the Cycle Threshold Value. Clin Infect Dis.

[34] Salvatore PP, Dawson P, Wadhwa A, Rabold EM, Buono S, Dietrich EA, Reses HE, Vuong J, Pawloski L, Dasu T, Bhattacharyya S, Pevzner E, Hall AJ, Tate JE, Kirking HL (2021). Epidemiological Correlates of Polymerase Chain Reaction Cycle Threshold Values in the Detection of Severe Acute Respiratory Syndrome Coronavirus 2 (SARS-CoV-2). Clin Infect Dis.

